# Amide Proton Transfer-Weighted MRI Might Help Distinguish Amnestic Mild Cognitive Impairment From a Normal Elderly Population

**DOI:** 10.3389/fneur.2021.707030

**Published:** 2021-10-12

**Authors:** Zixuan Guo, Yanchun Jiang, Xiaoyan Qin, Ronghua Mu, Zhuoni Meng, Zeyu Zhuang, Fuzhen Liu, Xiqi Zhu

**Affiliations:** ^1^Department of Medical Imaging, Guilin Medical University, Guilin, China; ^2^Department of Medical Imaging, Nanxishan Hospital of Guangxi Zhuang Autonomous Region, Guilin, China; ^3^Department of Neurology, Nanxishan Hospital of Guangxi Zhuang Autonomous Region, Guilin, China

**Keywords:** amide proton transfer imaging, Alzheimer's disease, amnestic mild cognitive impairment, hippocampus, amygdala, cortical-limbic circuit

## Abstract

**Objectives:** To evaluate whether 3D amide proton transfer weighted (APTw) imaging based on magnetization transfer analysis can be used as a novel imaging marker to distinguish amnestic mild cognitive impairment (aMCI) patients from the normal elderly population by measuring changes in APTw signal intensity in the hippocampus and amygdala.

**Materials and Methods:** Seventy patients with aMCI and 74 age- and sex-matched healthy volunteers were recruited for routine MRI and APT imaging examinations. Magnetic transfer ratio asymmetry (MTRasym) of the amide protons (at 3.5 ppm), or APTw values, were measured in the bilateral hippocampus and amygdala on three consecutive cross-sectional APT images and were compared between the aMCI and control groups. The independent sample t-test was used to evaluate the difference in APTw values of the bilateral hippocampus and amygdala between the aMCI and control groups. Receiver operator characteristic analysis was used to assess the diagnostic performance of the APTw. The paired t-test was used to assess the difference in APTw values between the left and right hippocampus and amygdala, in both the aMCI and control groups.

**Results:** The APTw values of the bilateral hippocampus and amygdala in the aMCI group were significantly higher than those in the control group (left hippocampus 1.01 vs. 0.77% *p* < 0.001; right hippocampus 1.02 vs. 0.74%, *p* < 0.001; left amygdala 0.98 vs. 0.70% *p* < 0.001; right amygdala 0.94 vs. 0.71%, *p* < 0.001). The APTw values of the left amygdala had the largest AUC (0.875) at diagnosis of aMCI. There was no significant difference in APTw values between the left and right hippocampus and amygdala, in either group. (aMCI group left hippocampus 1.01 vs. right hippocampus 1.02%, *p* = 0.652; healthy control group left hippocampus 0.77 vs. right hippocampus 0.74%, *p* = 0.314; aMCI group left amygdala 0.98 vs. right amygdala 0.94%, *p* = 0.171; healthy control group left amygdala 0.70 vs. right amygdala 0.71%, *p* = 0.726).

**Conclusion:** APTw can be used as a new imaging marker to distinguish aMCI patients from the normal elderly population by indirectly reflecting the changes in protein content in the hippocampus and amygdala.

## Introduction

Mild cognitive impairment (MCI) is the transitional stage between normal aging and dementia, in which cognitive function is slightly impaired, but daily living is not affected, and the condition has not yet reached the diagnostic criteria for dementia (1, 2). Although many efforts have been made to improve the treatment of Alzheimer's disease (AD) and dementia, little effect has been achieved. As a result, the research focus has gradually shifted to MCI, which is considered a pre-dementia stage. According to the impairment of specific cognitive domains, MCI can be further categorized as amnestic MCI (aMCI), presenting with impaired learning and memory functions, and non-amnestic MCI (naMCI), presenting with impaired cognitive domains (3, 4). MCI is often progressive, with aMCI having a higher risk of conversion to Alzheimer's disease and naMCI having a higher risk of conversion to non-Alzheimer's dementia. Approximately 5–10% of patients with MCI develop dementia each year, which is much higher than the annual incidence rate of 1 to 2% in the normal population (2, 5, 6). Usually, if treatment can be applied early in the course of the disease, impaired cognitive function is reversible (7).

AD imposes a severe burden on families and society, and the most effective treatment is to intervene in the early stages of AD (ideally at the aMCI stage) (8). Cognitive intervention for aMCI patients in the early stages of the disease can help to improve their overall cognitive function to a certain extent, and about 30% to 50% return to “normal” cognitive function in the subsequent follow-up (9). Therefore, the importance of early diagnosis is self-evident. It is believed that there is an overlap between normal brain aging and aMCI, and the difference between them may be very subtle (1). The prevalence of aMCI was 17.1% in a previous community-based elderly population in China (10). However, in community settings, the diagnosis and treatment of cognitive impairment by junior doctors at all levels are grossly inadequate, and more than half of such patients go undetected. Therefore, timely screening of this part of the population is very important (9). Currently, there is still a lack of effective biomarkers for identifying aMCI populations. It is believed that searching for an imaging biomarker may be a simple and effective solution for the early detection of aMCI.

Amide proton transfer (APT) imaging is a new type of magnetic resonance molecular imaging technology based on the signal generated by the proton exchange between the amide protons of mobile proteins and peptides in cells and free water and does not require external contrast agents. Since there are amide protons at +3.5 ppm, the magnetic transfer ratio asymmetry percentage (MTRasym) (3.5 ppm) (%) produced by asymmetric measurements at +3.5 ppm is defined as an APT weighted (APTw) image. As the temperature of the human body remains relatively constant, the APT effect depends primarily on the intracellular proteins and pH values (11, 12).

APT imaging is not limited to central nervous system tumors or ischemic lesions (13–16); more recently, it has been gradually developed and applied to the diagnosis of various system diseases (17–20), but its application in aMCI is still in its infancy. The limbic system is a set of brain structures involved in emotion, learning, and memory, which are central to cognitive function. The hippocampus and amygdala are part of the limbic system and are affected early on in aMCI and AD patients (21, 22). Autopsy results showed that the pathological features of aMCI were consistent with those of pre-AD (23). Abnormal protein deposition, including of extracellular amyloid plaques and intracellular neurofibrillary tangles (11, 24), increases the intracellular protein concentrations, ultimately leading to an increased APTw signal intensity. Zhang et al. (25) found differences in hippocampal APTw values between aMCI patients and the normal elderly and believed that the APTw signal could be used as an imaging marker in aMCI patients. However, the sample size was too small. Sartoretti et al. (26) reported that there were significant differences in APTw signal intensity between the two hemispheres of the brain, and the overall APTw value on the left side of the brain was higher than that on the right side. Wang et al. (24) found that APTw signals in the hippocampus of patients with AD were significantly higher than those in normal subjects, but aMCI patients were not involved. All of the above studies ignored the functional connection between the hippocampus and amygdala. Both the hippocampus and amygdala are central parts of the cortical-limbic circuit (27), and direct nerve fiber connections run between them (28). In the case of cognitive decline, changes in APTw signals in the hippocampus and amygdala may occur simultaneously. Therefore, we assume that the APTw values of the hippocampus and amygdala of aMCI patients are significantly higher than those of normal elderly people and that these APTw values can be used as a quantifiable imaging marker to effectively identify aMCI patients in the early stages.

Based on this hypothesis, the aim of this study was to investigate whether APTw values in the hippocampus and amygdala differ between aMCI patients and the normal elderly population. To explore whether the APTw values of the left hippocampus and amygdala are different from those of the right hippocampus and amygdala in the same individual, both aMCI patients and normal elderly individuals were enrolled.

## Materials and Methods

### Subjects

From October 2020 to February 2021, 80 patients with aMCI and 90 healthy volunteers were recruited (11 subjects were excluded from the study because they could not cooperate with normal examination; 15 subjects were excluded from the study because their image quality did not meet the diagnostic requirements). Finally, 70 patients with aMCI and 74 healthy volunteers were enrolled, and all subjects were assigned a label that did not identify the group assignment. The sex, age, education, and hippocampal (amygdala) volume of all subjects were collected to avoid bias in the results caused by differences in these parameters between the two groups. The test to verify the inclusion criteria for aMCI patients were carried out by an experienced neurological physician, according to the DSM-IV and Petersen diagnostic criteria, which included (i) cognitive impairment reported by patients, insiders, or experienced clinicians and (ii) objective evidence of impairment in one or more cognitive regions and poor performance in episodic memory from cognitive tests. According to the recommendations of relevant research (29, 30), we used the Montreal Cognitive Assessment (MoCA), which is more sensitive and more suitable for Chinese elderly communities, to replace the Mini-Mental State Examination (MMSE) in the evaluation of cognitive function, thereby increasing the detection rate of aMCI patients. The MOCA scores of aMCI patients were ≤13 in the illiteracy group, ≤19 in the elementary school group, and ≤24 in the middle school and above group, with HIS <4 points) (29); (iii) complex instrumental daily ability can be slightly impaired, but independent daily living abilities are retained; (iv) the diagnostic criteria for dementia have not been met; and v) right-handed. Exclusion criteria were as follows: (i) serious medical or mental illness; 9ii) neurological diseases (such as hydrocephalus, brain tumor, cerebrovascular malformation, Parkinson's disease, previous large-scale cerebral infarction, traumatic brain injury, etc.); (iii) a history of psychotropic drug use or alcohol abuse; and (iv) patients with MRI contraindications, or an inability to complete neurological scale examination. The healthy control group was evaluated using the MOCA, and no obvious cognitive impairment was found in any participants. All participants were right-handed. An informed consent form was obtained from all subjects. This study was approved by the Ethics Committee of our hospital (Nanxishan Hospital, Guangxi Zhuang Autonomous Region) (2020NXSYYEC-006). The basic demographic and neuropsychological data are presented in Table 1.

**Table 1:** Demographics characteristics and psychometric measures.

**Characteristic**	**aMCI (*N* = 70)**	**Healthy control (*N* = 74)**	**Statistics**	***p* value**
Age, y	54.47 ± 7.82	52.28 ± 5.97	1.879	0.062
Male/female	24/46	28/46	0.197	0.657
Education, y	8.71 ± 3.40	12.34 ± 4.14	−5.755	< 0.001
MoCA	19.79 ± 3.83	26.05 ± 2.04	−12.160	< 0.001
Right-hippocampus corrected volume, mm^3^	3485.58 ± 773.19	3708.04 ± 583.05	−1.941	0.054
Left-hippocampus corrected volume, mm^3^	3599.46 ± 750.00	3796.19 ± 618.16	−1.712	0.089
Right-amygdala corrected volume, mm^3^	1444.06 ± 321.55	1500.83 ± 302.94	−1.110	0.269
Left-amygdala corrected volume, mm^3^	1450.96 ± 332.56	1527.68 ± 295.93	−1.464	0.145

*Two-sample t-test for all comparisons except sex, where Pearson χ^2^-test was used. MoCA, Montreal Cognitive Assessment; aMCI, amnestic mild cognization impairment*.

### MRI Data Acquisition

In our study, a 3.0-T (Ingenia 3.0CX; Philips Healthcare, Best, The Netherlands) magnetic resonance imaging system and 16-channel coils of the head were used to perform MRI transection scans. Scan sequence: 3D T1W FFE, TR = 6.4 ms, TE = 3.0 ms, FOV = 240 × 240 × 180 mm, reconstruction voxel = 1.1 × 1.1 × 1.1, reconstruction matrix = 400 × 400, slice thickness = 1.1 mm; 3D T2 SE, TR = 2,500 ms, TE = 232 ms, FOV = 250 × 25 × 180 mm, reconstruction voxel = 1.1 × 1.1 × 1.1, reconstruction matrix = 512 × 512, slice thickness = 1.1 mm; 3D FLAIR, TR = 4,800 ms, TE = 244 ms, FOV = 240 × 240 × 173 mm, reconstruction voxel = 1.1 × 1.1 × 1.1, reconstruction matrix = 384 × 384, slice thickness = 1.2 mm; 3DAPTw sequence, TR = 6,300 ms, TE = 8.3 ms, FOV = 230 × 180 × 60 mm, reconstruction voxel = 1.8 × 1.8 × 6, reconstruction matrix = 256 × 256, slice thickness = 6 mm, TSE factor = 174. Each subject underwent the above MRI scan regimen, which took approximately 30 min.

### Data Processing

**Figure 1:**
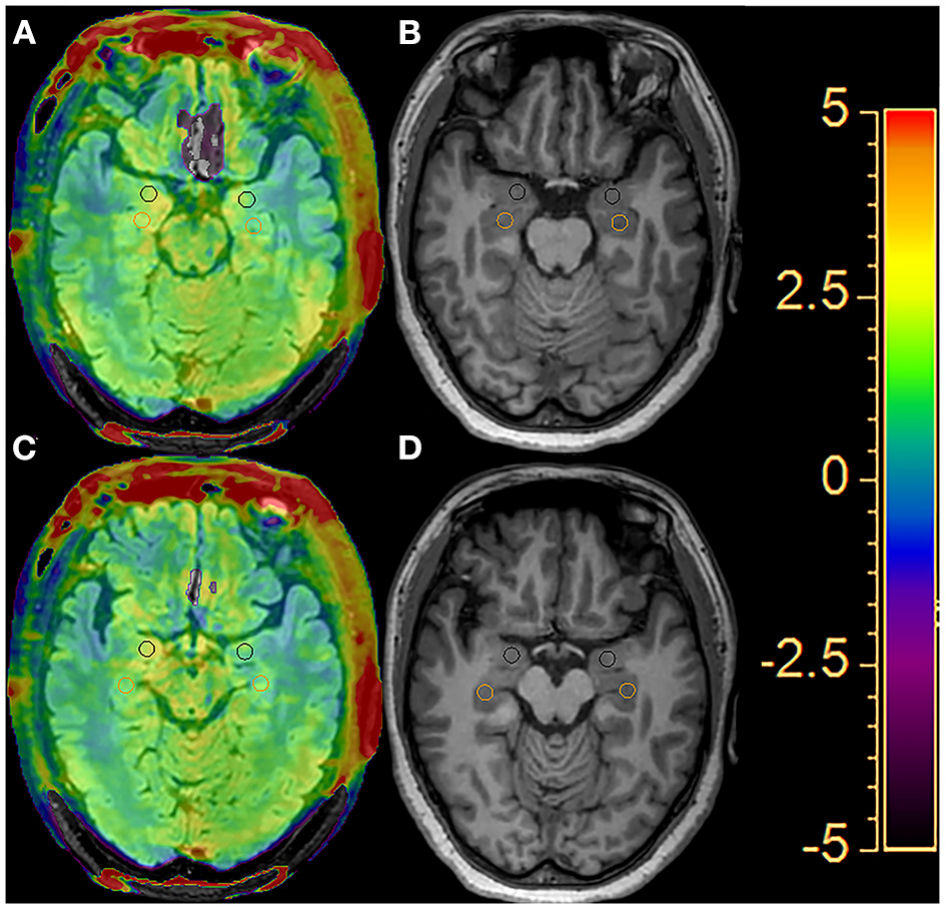
An example of the definition of the regions of interest (ROIs) of the bilateral hippocampus (orange circle) and amygdala (black circle) for quantitative analyses. Two consecutive layers of the FLAIR image with APTw overlay **(A, C)** and the T1-weighted image **(B, D)**.

The APTw images were generated directly by the host according to the scanning scheme. APTw images were co-registered and overlaid with geometrically identically acquired FLAIR images on a dedicated workstation “IntelliSpace Portal” version 8 (Philips Healthcare, Best, the Netherlands). The regions of interest (ROIs) were outlined on the fused image by a radiologist with 15 years of experience in neuroradiology who was blinded to the group assignment. ROI outline criteria are as follows: (i) the circular measurement tool was used to delineate the two consecutive layers in the hippocampus and amygdala and to calculate the average value; (ii) the size of the bilateral ROI of all subjects remained basically the same, with an area of about 30 mm^2^; and (iii) avoid areas of cystic change, necrosis, and bleeding (Figure 1). The APTw(%) calculation method was as follows (12):


$$\begin{array}{l} MTRasym\left(\Delta \omega \right)\left(\%\right)=100{\%}^{*}\left(S-\Delta \omega -S+\Delta \omega \right)÷S0.\\ \text{When }\Delta \omega \;=+\;3.5\text{ ppm},\text{ one has}: \end{array}$$

$$\begin{array}{l} \text{APTw}(\%)\text{=MTRasym}(\text{3}.5\text{ ppm})(\%)=\text{100}{\%}^{*}(\text{S}-\text{3}.\text{5 ppm}\\ -\text{ S}+\text{3}.\text{5 ppm})÷\;\text{S0}. \end{array}$$

### Volume Data Processing

On the oblique coronal plane of the 3D T1W FFE sequence, the area of each layer of the hippocampus (amygdala) was manually sketched and added together, then multiplied by the layer thickness, and the original volume of the unilateral hippocampus (amygdala) was obtained. To eliminate the effect of individual differences in cranial size on volume data, the original volumes of the hippocampus and amygdala were corrected. The anteroposterior, transverse, and axial diameters of the cranial cavity were measured and multiplied to obtain the volume. The anteroposterior diameter was defined as the distance from the frontal pole to the occipital pole in the median sagittal images. The transverse diameter was defined as the maximum transverse diameter between the intralaminae perpendicular to the anteroposterior diameters on the horizontal transverse section of the dorsal thalamus. The axial diameter was defined as the distance between the intracranial plates from the anterior inferior border of the foramen magnum to the cranial crest on the median sagittal image. The correction formula was


$$\begin{array}{l} {\text{V}}^{\prime }=\text{V}\times \text{V mean/Vn}. \end{array}$$

where V′ is the corrected volume of the hippocampus or amygdala of a single subject, V is the original volume of the hippocampus or amygdala of a single subject, Vmean is the average volume of the cranial cavity of all subjects, and Vn is the volume of the cranial cavity of a single subject. To avoid the effect of subjective factors on manual delineation of the hippocampal and amygdala boundaries, the surveyor was unaware of the subject's clinical data and grouping.

### Statistical Analysis

SPSS software (version 21.0) was used to perform the statistical analyses. After testing for normality, the chi-square test and independent sample *t*-test were applied to analyze the statistical differences in the demographic, clinical, and quantitative imaging parameters between the aMCI and control groups. The APTw values of the bilateral hippocampus and amygdala between the aMCI and control groups were compared using an independent sample *t*-test. A paired *t*-test was used to assess the difference in APTw values between the left and right hippocampus and amygdala, in both the aMCI group and control group. Receiver operator characteristic (ROC) curves were used to assess the diagnostic performance of the APTw values at each ROI. The bilateral volume and APTw values of the hippocampus and amygdala, age, and MoCA scores of the aMCI and control groups were all expressed as *x* ± *s*. Statistical significance was set at *p* < *0.05*. All figures were generated using GraphPad Prism 9.0 (GraphPad Software Inc., San Diego, CA, USA).

## Results

### Patient Demographics

There was no significant difference in age, education, sex, bilateral amygdala volume, or bilateral hippocampal volume between the aMCI patients and normal subjects. However, compared with the healthy controls, the average MoCA scores were significantly lower in the aMCI group, as shown in Table 1.

### Comparison of APTw Values in the Hippocampus and Amygdala Between AMCI and Healthy Control

**Figure 2:**
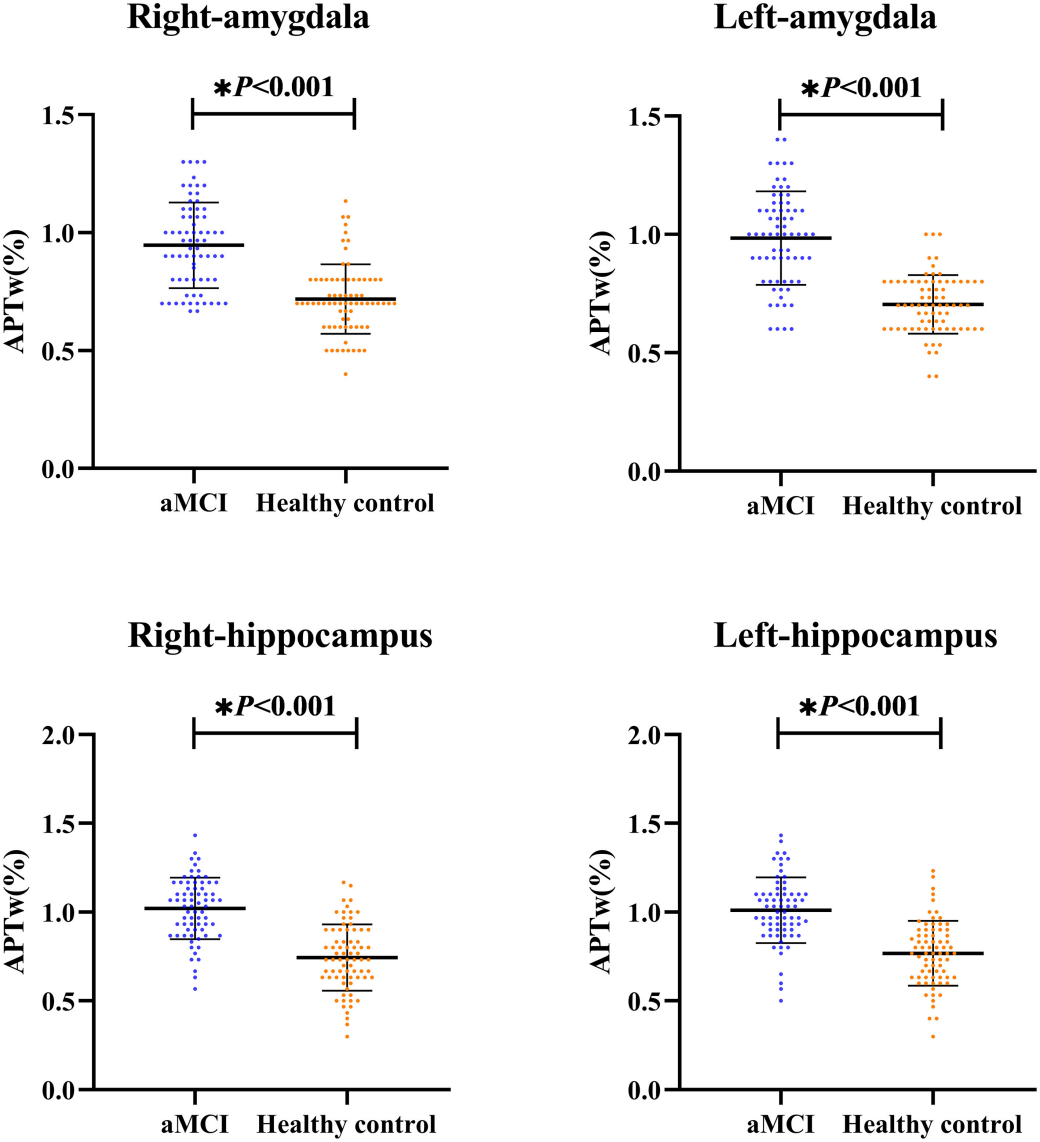
The independent sample *t*-test for the APTw values. The results are shown from the regions of interest (ROIs) in aMCI groups (blue) and the healthy control (orange). * The difference was significant.

As shown in Figure 2 and Table 2, the APTw images of the aMCI patients demonstrated higher APTw intensity values in the bilateral hippocampus and amygdala, compared to the healthy control (right hippocampus *p* < 0.001; left hippocampus *p* < 0.001; right amygdala *p* < 0.001; left amygdala *p* < 0.001).

**Table 2:** Comparison of APTw values between aMCI and healthy control (%).

	**aMCI (N = 70)**	**Healthy control (*N* = 74)**	**t**	***p* value**
Right hippocampus	1.02 ± 0.17	0.74 ± 0.19	9.176	<0.001^*^
Left hippocampus	1.01 ± 0.19	0.77 ± 0.18	7.948	<0.001^*^
Right amygdala	0.94 ± 0.18	0.71 ± 0.14	8.811	<0.001^*^
Left amygdala	0.98 ± 0.18	0.70 ± 0.12	10.163	<0.001^*^

* *The difference was significant; aMCI, amnestic mild cognization impairment*.

### Comparison of APTw Values of Both Sides

**Figure 3:**
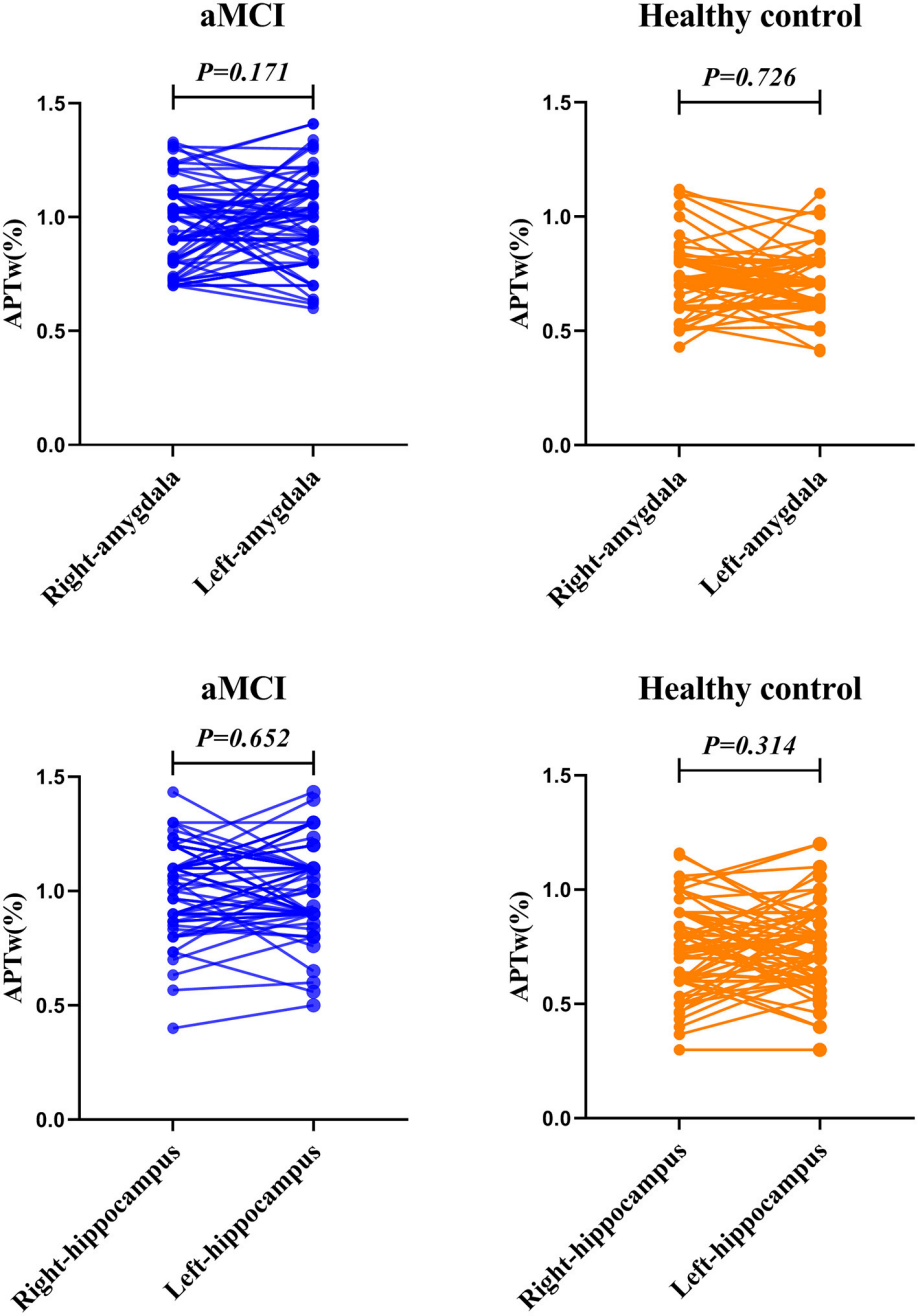
The paired *t*-test for the APTw value of the bilateral amygdala and hippocampus. The results are shown from the regions of interest (ROIs) in aMCI (blue) group and the healthy control (orange).

Figure 3 and Table 3 compare the APTw values of the bilateral hippocampus and amygdala in the different groups. We found that there was no significant difference in APTw values between the left and right hippocampus and amygdala, in either the aMCI or the control group (aMCI group left hippocampus 1.01 vs. right hippocampus 1.02%, *p* = 0.652; healthy control group left hippocampus 0.77 vs. right hippocampus 0.74%, *p* = 0.314; aMCI group left amygdala 0.98 vs. right amygdala 0.94%, *p* = 0.171; healthy control group left amygdala 0.70 vs. right amygdala 0.71%, *p* = 0.726).

**Table 3:** Comparison of APTw values on both sides (%).

	**Right side**	**Left side**	***t***	***p* value**
Hippocampus in aMCI	1.02 ± 0.17	1.01 ± 0.19	0.453	0.652
Hippocampus in healthy control	0.74 ± 0.19	0.77 ± 0.18	−1.014	0.314
Amygdala in aMCI	0.94 ± 0.18	0.98 ± 0.18	−1.383	0.171
Amygdala in healthy control	0.71 ± 0.14	0.70 ± 0.12	0.352	0.726

*aMCI, amnestic mild cognization impairment*.

### Accuracy of APTw MRI in Diagnosing aMCI

**Figure 4:**
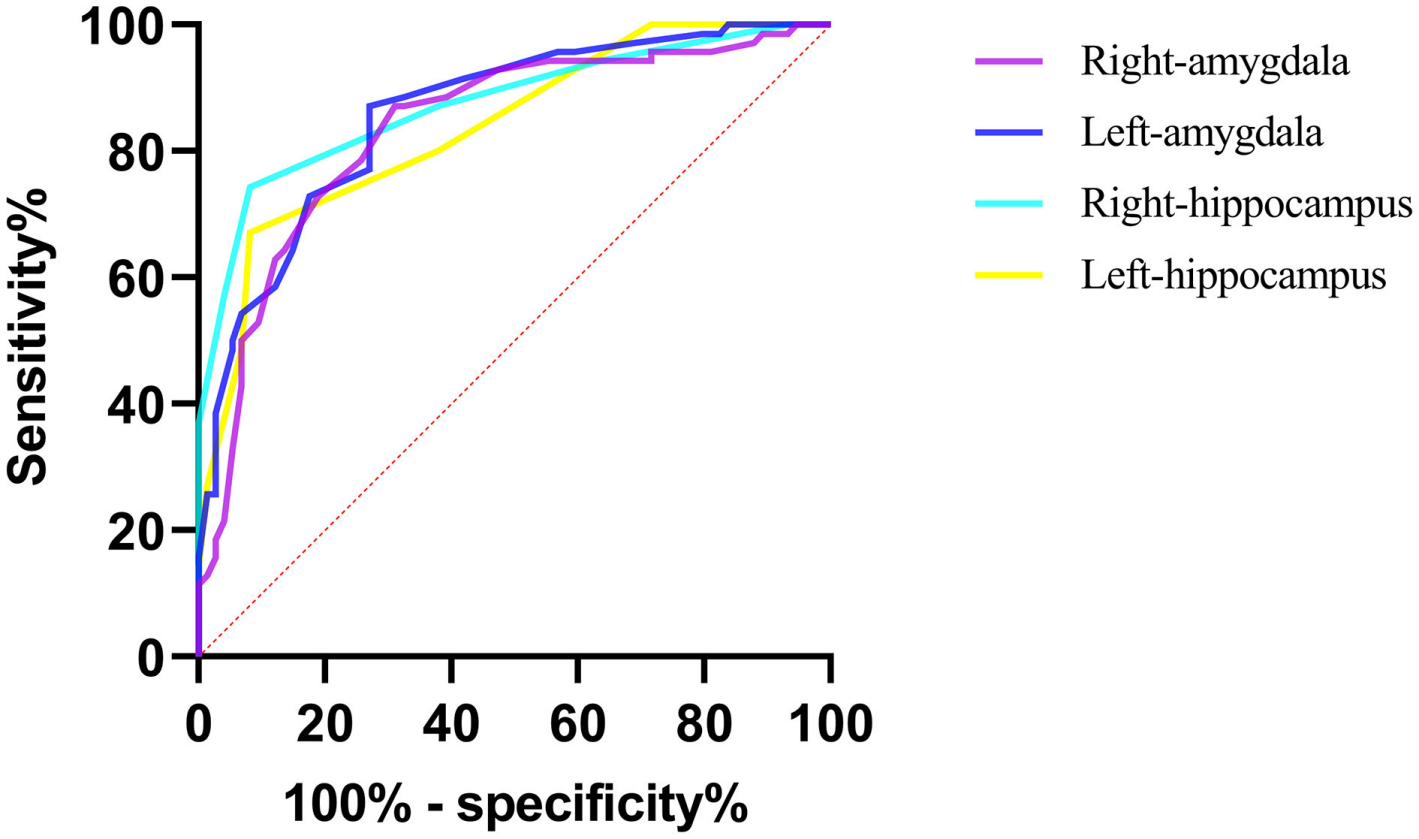
ROC curves showed the ability of the bilateral hippocampus and amygdala APTw values to diagnose aMCI patients.

The diagnostic performances of these four ROIs are shown in Figure 4. All four ROIs showed significantly higher APTw values in aMCI patients than in healthy controls. ROC curve analysis showed that APTw values have great potential as new imaging biomarkers for the diagnosis of aMCI patients. The largest areas under the ROC curves (Table 4) were 0.875 in the left amygdala (with a 74.3% sensitivity and a 91.9% specificity at the cutoff APTw signal intensity of 0.850%), 0.858 in the right hippocampus (with 87.1% sensitivity and 73.0% specificity at the cutoff APTw signal intensity of 0.842%), 0.836 in the left hippocampus (with 87.1% sensitivity and 68.9% specificity at the cutoff APTw signal intensity of 0.858%), and 0.838 in the right amygdala (with a 67.1% sensitivity and a 91.9% specificity at the cutoff APTw signal intensity of 0.850%).

**Table 4:** Diagnostic performance of APTw values from four regions of interest (ROIs) in predicting aMCI.

**ROIs**	**AUC; [95% CI]**	**Cutoff value (%)**	**Sensitivity (%)**	**Specificity (%)**
Right hippocampus	0.858; [0.799, 0.918]	0.842	87.1	73.0
Left hippocampus	0.836; [0.769, 0.903]	0.858	87.1	68.9
Right amygdala	0.838; [0.774, 0.902]	0.850	67.1	91.9
Left amygdala	0.875; [0.817, 0.933]	0.850	74.3	91.9

*APTw, amide proton transfer-weighted; aMCI, amnestic mild cognitive; AUC, area under the ROC (receiver operator characteristic) curve; CI, confidence interval*.

## Discussion

Our study found that the bilateral APTw values of the hippocampus and amygdala were significantly higher in aMCI patients than those in the normal elderly population, consistent with the results of Zhang et al. (25). The ROC curve results showed that all APTw values had good diagnostic performance, among which the APTw values of the left amygdala had the best diagnostic performance. We also found no significant differences in the APTw values between the left and right hippocampus and amygdala, in either the aMCI or control group.

APT imaging is a protein content- and pH-dependent MRI technique (11, 12). Compared with the pH value (33%), the intracellular mobile protein content (66%) contributes more to the APT signal (31, 32). It is well known that the concentration of mobile proteins and semisolid macromolecules in brain tissues increases with age during normal brain aging, but the deposition of abnormal proteins in the hippocampus in patients with aMCI and AD results in the APTw values being significantly higher than those in normal elderly people of the same age (24, 33). Since there were no significant differences in temperature or pH values between the brains of aMCI patients and the normal elderly population, the APTw values almost completely represent the differences in amide proton concentration. Our results thus indicate that the hippocampus and amygdala regions of aMCI patients indeed have higher amide proton levels as a result of the deposition of abnormal proteins. ROC curves showed that all the bilateral APTw values of the hippocampus and amygdala had a good diagnostic performance at identifying aMCI populations from healthy populations. The APTw values of the left amygdala yielded the highest diagnostic performance, with an AUC of 0.875. It has been speculated that the accumulation of abnormal proteins and harmful substances in the brain of aMCI patients may damage the neurons and lead to death, while APTw signals are generated by living tissues (11). Therefore, the APTw values will rise to a certain level and then decrease with neuronal death, eventually reaching a balance with the APTw values of normal elderly individuals (25). Another study (24) suggested that hippocampal APTw values increased continuously from the normal elderly to severe AD. Further research is needed to verify these findings. Both the hippocampus and amygdala are central parts of the cortico-limbic circuit (27). We believe that the cortico-limbic circuit in aMCI patients has been impaired and that cortico-limbic dysfunction may play an important role in the decline of cognitive function in aMCI and AD patients.

Consistent with the study of Dreher et al. (34), we found no significant difference in mean APTw values between the left and right hippocampus and the amygdala in healthy older adults (left hippocampus 0.77 vs. right hippocampus 0.74%, *p* = 0.314; left amygdala 0.70 vs. right amygdala 0.71%, *p* = 0.726). However, by comparing APTw values in 22 different regions of the cerebral hemisphere between 20 healthy young volunteers, Sartoretti et al. (26) found that APTw values in the left hippocampus and amygdala were significantly higher than those in the right (hippocampal body 1.39 vs. 1.2%; amygdala 1.57 vs. 1.34%), which is inconsistent with our results. First, we hypothesized that this might be related to the size of the sample; our sample size was 74, compared with 20 for Sartoretti et al. Second, the effect of age on the APTw value cannot be ignored (33). The subjects of our study were middle-aged and elderly healthy people, while the subjects of Sartoretti et al. (26) were young healthy volunteers. However, recent studies have shown no significant correlation between APT signals and age in any brain regions (35). In addition, the APTw values in both the hippocampus and amygdala were significantly higher in the study by Satoretti et al. (26) in healthy Europeans. Since the subjects in our study were all healthy Chinese, we compared two other studies of APTw values in the hippocampus of Chinese subjects (24, 25) and found that the APTw values in the hippocampus in these studies were similar to our results. Therefore, we suspect that this may be related to ethnic differences. To test our hypothesis, a large epidemiological survey is required. We also compared the APTw values of the left and right hippocampus (amygdala) in the aMCI population and found no statistical difference, which was consistent with the findings in the normal aging people (left hippocampus 1.01 vs. right hippocampus 1.02%, *p* = 0.652; left amygdala 0.98 vs. right amygdala 0.94%, *p* = 0.171).

To the best of our knowledge, this is the first study focusing on the difference in APTw values of the hippocampus and amygdala in both sides of aMCI patients. We concluded that there is no significant difference in APTw values of the bilateral hippocampus (amygdala) in either the aMCI patients or healthy subjects. However, there is an objective difference in the volume of gray matter between the left and right hemispheres; i.e., there are different numbers of neurons and hemispheric connections in the left and right hemispheres (36). We speculate that the differences in the number of neurons in the hippocampus and amygdala on both sides are not large enough, and thus the differences in concentration of amide protons and peptides are too small to cause a change in the APT signal.

Because neurological scales are unstable and require a high degree of patient cooperation, we attempted to use APT as a more objective and stable imaging biomarker to assist clinicians in making a timely diagnosis of patients with aMCI who could not cooperate with the scale examination. Compared with previous studies (25, 26, 34), we used 3D APT imaging technology with a larger sample size. Finally, we obtained the same results, suggesting that the APTw values can be used as a new and promising imaging biomarker for aMCI diagnosis. However, as a new magnetic resonance imaging technology, APT imaging is not mature and has many defects. APT magnetic resonance imaging is prone to confounding signals that can lead to misdiagnosis. For example, hippocampal measurements may be affected by the surrounding cerebrospinal fluid (24). The MT and rNOE effects have also been proven to interfere with the APT signal. Meanwhile, B0 inhomogeneity is a key problem in APT imaging (11). All these factors may restrict the application of APT in aMCI.

This study has several limitations. First, our sample size was relatively small, and the study was performed with a cross-sectional design. Before applying APT to aMCI, a longitudinal study with a larger sample size is needed in the future. Second, the differentiation of MCI subtypes is of great significance for the prevention and treatment of dementia (4). This study only compared the difference in APTw values between aMCI patients and normal elderly people but did not involve naMCI patients. Furthermore, freehand ROI analysis and volume measurement can produce artificial errors, which might affect the accuracy of the APTw values. Finally, this study is based on the Chinese community population, and therefore, the results may not be applicable to other ethnic groups due to ethnic and cultural differences.

## Conclusion

In conclusion, in this study, the APT technique was used to reflect the differences in the protein content of the bilateral hippocampus and amygdala between aMCI patients and the normal elderly population and verified the possibility of using APTw values as an imaging marker of aMCI. We have demonstrated that the novel APT MRI technology has great potential in the diagnosis of neurodegenerative diseases. However, more studies and experiments are needed to explore the potential of APT MRI technology in the future.

## Data Availability Statement

The raw data supporting the conclusions of this article will be made available by the authors, without undue reservation.

## Ethics Statement

The studies involving human participants were reviewed and approved by Ethics Committee of Nanxishan Hospital, Guangxi Zhuang Autonomous Region. The patients/participants provided their written informed consent to participate in this study.

## Author Contributions

ZG and XZ designed the study. ZG, XQ, RM, and ZM conducted the MRI data processing and statistical analyses. ZM, RM, ZZ, and FL contributed to data collection and analyses. ZG, RM, and ZM wrote the paper. XZ and YJ critically revised the manuscript. All authors approved the final draft.

## Funding

This study was sponsored by the Project of Health Commission of Guangxi Zhuang Autonomous Region (Z20190997, Z20191023), Social Development Program of Guilin Science and Technology Bureau (2020011206-7), Guangxi Zhuang Autonomous Region Nanxishan Hospital Excellence Program (NY2019003), and Graduate Student Innovation Program of Guilin Medical University.

## Conflict of Interest

The authors declare that the research was conducted in the absence of any commercial or financial relationships that could be construed as a potential conflict of interest.

## Publisher's Note

All claims expressed in this article are solely those of the authors and do not necessarily represent those of their affiliated organizations, or those of the publisher, the editors and the reviewers. Any product that may be evaluated in this article, or claim that may be made by its manufacturer, is not guaranteed or endorsed by the publisher.

## Abbreviations

AD: Alzheimer's disease
aMCI: amnestic mild cognitive impairment
APTw: amide proton transfer-weighted
MRI: magnetic resonance imaging
ROI: region of interest
MoCA: Montreal Cognitive Assessment
ROC: receiver operator characteristic
AUG: area under curve.
